# Electrocautery-Assisted Management of Unilateral Pyogenic Granuloma: A Case Report

**DOI:** 10.7759/cureus.57794

**Published:** 2024-04-07

**Authors:** Unnati Shirbhate, Pavan Bajaj, Aayushi Pakhale, Khushboo Durge, Ranu Oza, Shivani Thakre

**Affiliations:** 1 Department of Periodontics, Sharad Pawar Dental College, Datta Meghe Institute of Higher Education and Research, Wardha, IND; 2 Department of Oral Pathology, Sharad Pawar Dental College, Datta Meghe Institute of Higher Education and Research, Wardha, IND

**Keywords:** electrosurgical unit, soft-tissue lesion, tumour-like growth, histopathology, excision, electrocautery, gingival enlargement, pyogenic granuloma

## Abstract

Pyogenic granuloma (PG) refers to an acquired benign proliferation most commonly seen within the oral cavity involving lips, palate, and gingiva. The term is misleading since it is a type of lobular capillary haemangioma but not an infection. It frequently recurs but lacks the capacity for malignant alteration. Depending on where the PG is located, one may experience discomfort or irritation. PGs often lead to differential diagnoses by clinicians, which include capillary hemangioma, neurofibroma, melanoma, and hyperplasia. Therefore, one must confirm a PG by diagnosing and analysing it by clinical and histopathological examinations, and treatment options should be formulated according to the evaluation. Sometimes, a biopsy of the lesion can be taken for final diagnosis. Various treatment approaches are available, including conventional scalpel excision, laser, electrocautery, and cryotherapy. Surgical excision is preferable due to the likelihood of malignancy, as it provides the best cosmetic appearance and produces a specimen for pathologic assessment. After confirming all the clinical evaluatory parameters and routine haematological examinations, which proved satisfactory and within normal ranges, this case of a 45-year-old female with soft tissue growth of the gingival origin was managed by electrocautery, and the PG was confirmed by a clinical-histopathological examination.

## Introduction

A reactive proliferation of connective tissue from local irritants is known as a pyrogenic granuloma (PG). It is a tumour-like development of the oral cavity, thought to be neoplastic. It is typically found around the anterior teeth or gingival margin. The term PG can be deceptive because the lesion does not contain pus and is not technically a granuloma [1]. In 1904, Hartzell coined the term "pyogenic granuloma." The term "hemangiomatous granuloma," which was introduced by Angelopoulos, appropriately describes the inflammatory nature of oral pyogenic granuloma and its histopathologic appearance (hemangioma-like) [2]. While PGs can affect anyone at any age, it is most common in children and young adults between the ages of 11 and 40, with over 70% of cases involving females. The vascular effects of female hormones are the source of the preference towards women. As a PG rarely alleviates on its own and frequently bleeds heavily and repeatedly, surgery usually becomes necessary [1,2].

It is assumed that the primary etiologic component is unrelated to infection despite pyogenic organisms being suspected of causing it. Gingiva is reported to be affected mainly by PG. In addition to gingiva, the lips, tongue, buccal mucosa, and palate may be impacted. PGs are typically deep red to reddish-purple gingival masses that are soft, painless, pedunculated, or sessile [3]. According to Bhaskar and Jacoway, 1.85% of all oral diseases are caused by PGs [4]. Excision is the most common treatment for PGs and has the lowest recurrence rate. Alternative treatment options include curettage, traditional scalpel technique, electrocautery, radiosurgery, cryosurgery, sclerotherapy, and laser treatment, depending on the patient's need and the size of the lesion, where large lesions are managed by surgical excision approaches [5].

## Case presentation

**Figure 1:**
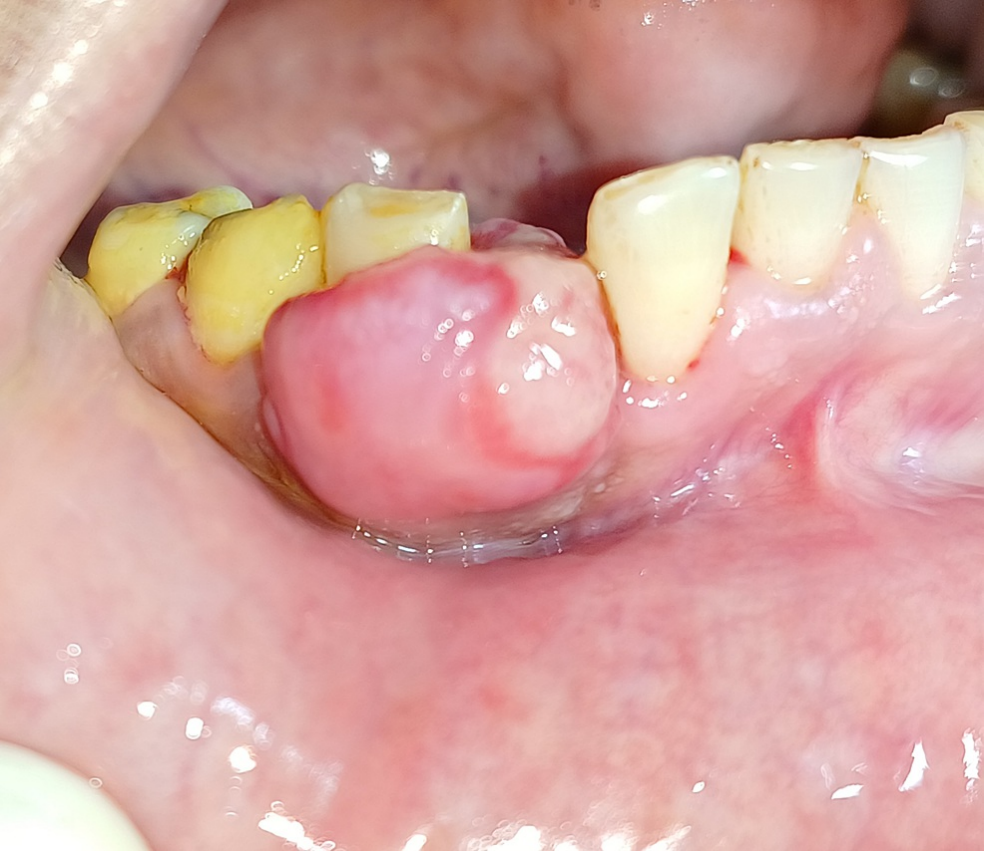
Pre-operative view after oral prophylaxis of the pyogenic granuloma lesion which was soft, pedunculated and edematous in origin

A 45-year-old female patient reported to the Department of Periodontics with a complaint of soft tissue growth associated with the lower right anterior region of the jaw for seven months. Past medical history and dental history were non-significant. A clinical examination revealed soft tissue growth, which was edematous, pedunculated, and unilateral, which was present on the facial and lingual aspect of the lower right lateral incisor and canine, as appreciated in Figure 1.

**Figure 2:**
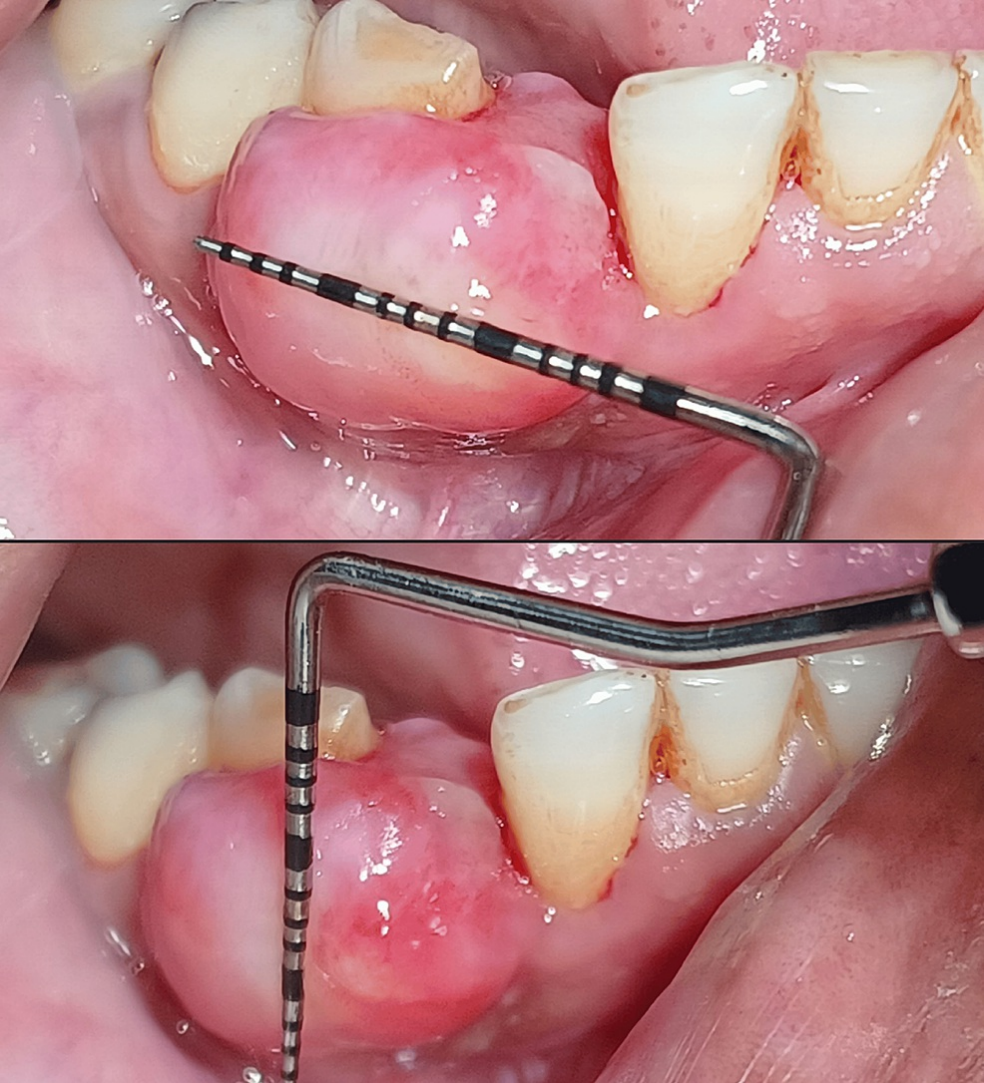
Measurements of the lesion were obtained with a University of North Carolina (UNC)-15 probe of width and height 13 mm and 11 mm, respectively

The lesion was insidious in onset and gradually progressive, having a size of 13 mm in width and 11 mm in height, as shown in Figure 2, and was painless, but pain aggravated on mastication and brushing. Oral prophylaxis was carried out before attempting the surgical procedure.

**Figure 3:**
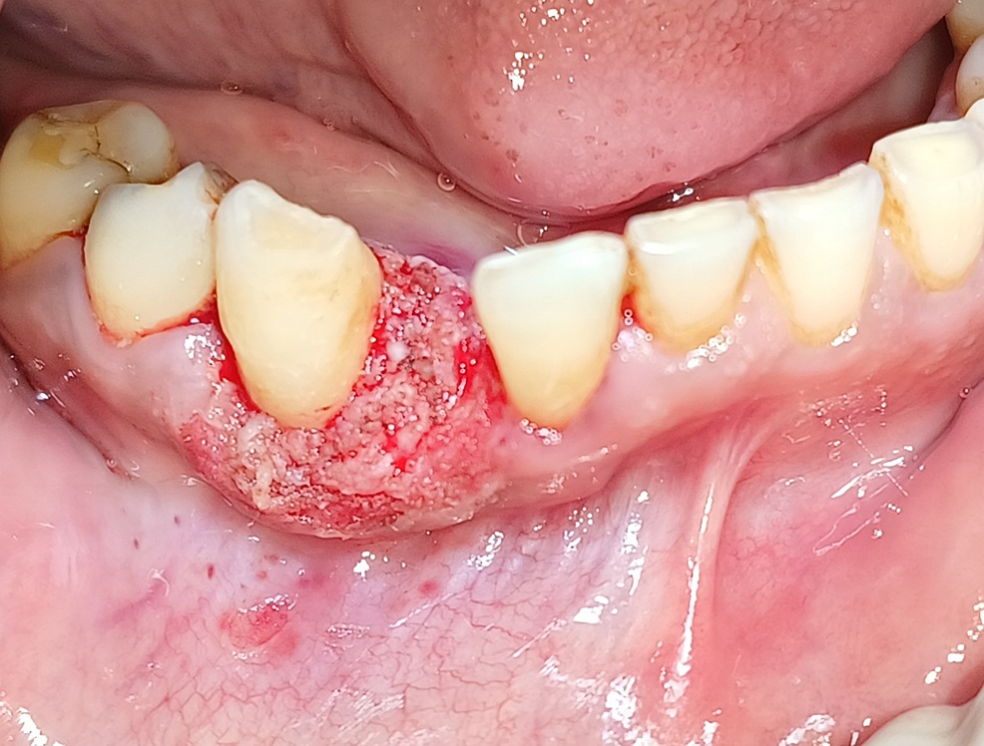
Surgical excision of the lesion was carried out with the electrosurgical unit

The patient was advised surgical excision of the lesion. Informed written consent was obtained. After one week of oral prophylaxis, surgical excision intervention was carried out using electrocautery. Under local anaesthesia, the lesion was excised with the electrosurgical unit setting on the cutting electrode, which was set to a power supply of 230V and frequency of 50Hz, 1.25 A, with power output kept at 50W, as seen in Figure 3.

**Figure 4:**
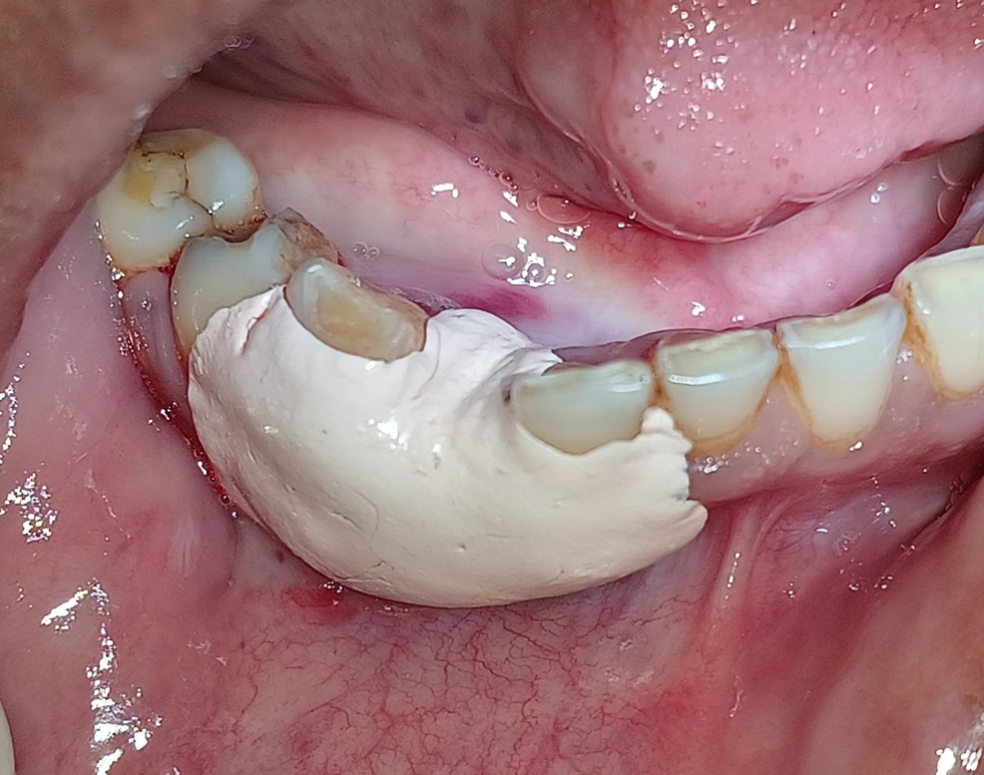
Periodontal pack was administered after excision

Hemostasis was achieved, and a periodontal pack was administered at the surgical site, as shown in Figure 4. The patient was instructed about the postoperative instructions and medication, with recall after seven days for pack removal.

**Figure 5:**
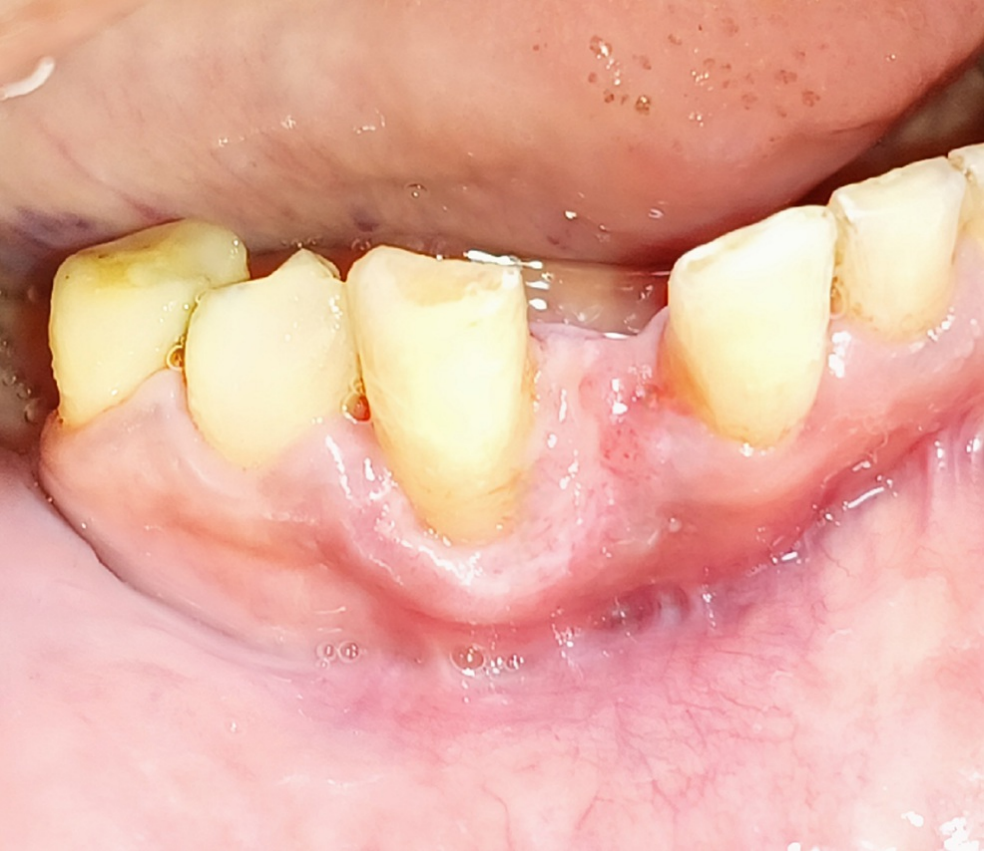
Post-operative view after seven days showing complete satisfactory healing of the lesion

Patients were reviewed after seven days for pack removal and after three months from the date of surgery. After seven days of follow-up examination, satisfactory healing of the surgical site and maintaining aesthetics at the concerned area with no pain and discomfort is appreciated in Figure 5.

**Figure 6:**
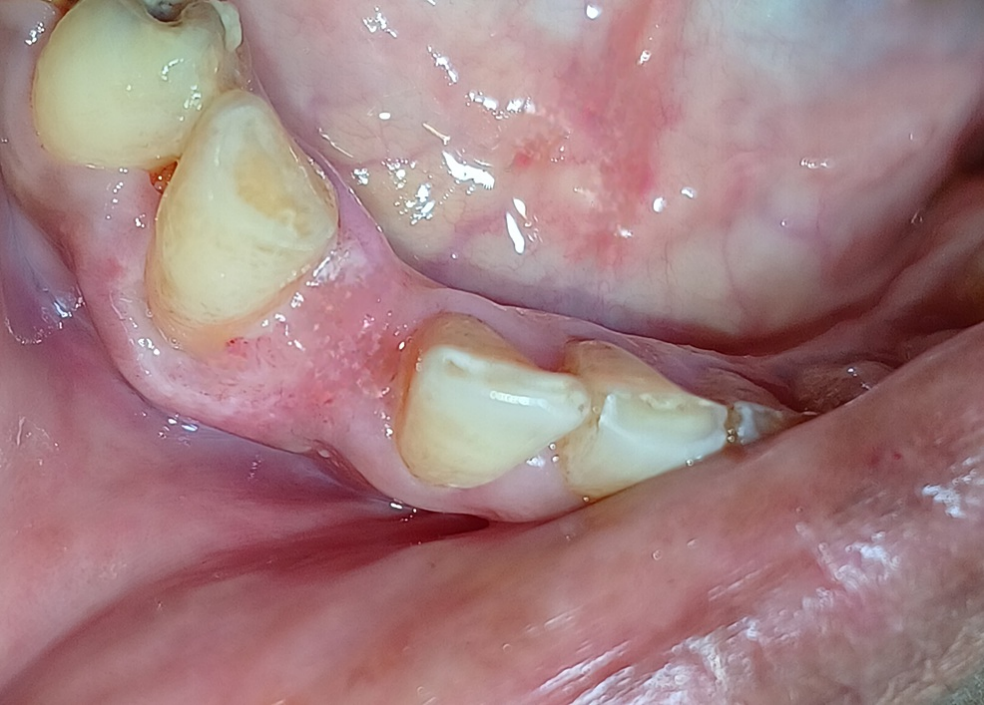
Postoperative view of the lesion after three months showing no recurrence and completely satisfactory healing

A follow-up examination after three months showed no recurrence of the lesion, no scar formation, and completely satisfactory healing, as appreciated in Figure 6.

**Figure 7:**
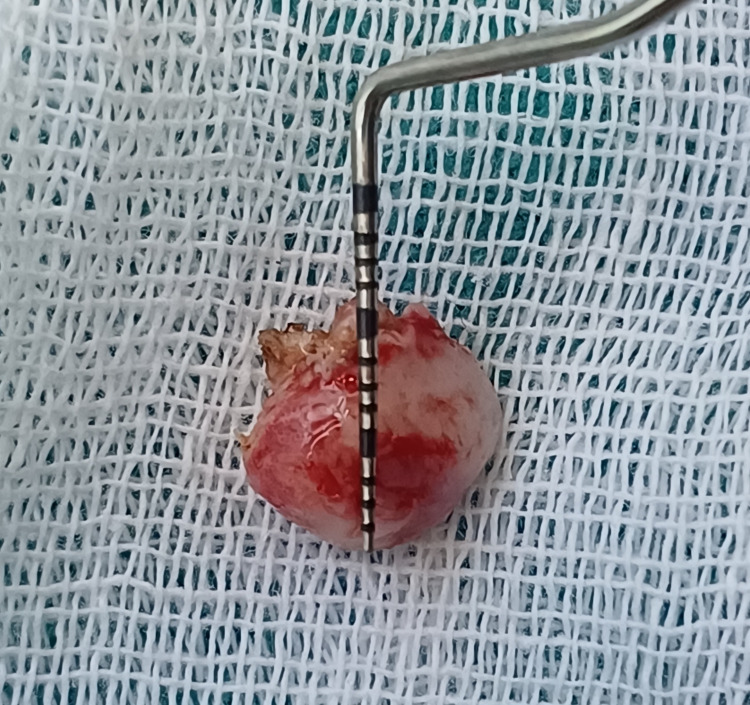
Excised specimen of the lesion, which was sent for a histopathological examination

The excised tissue of the lesion of the surgical site was sent in a sterile container for histopathological examination in a sterile saline solution, which confirmed the final diagnosis of a PG associated with gingiva, as shown in Figure 7.

**Figure 8:**
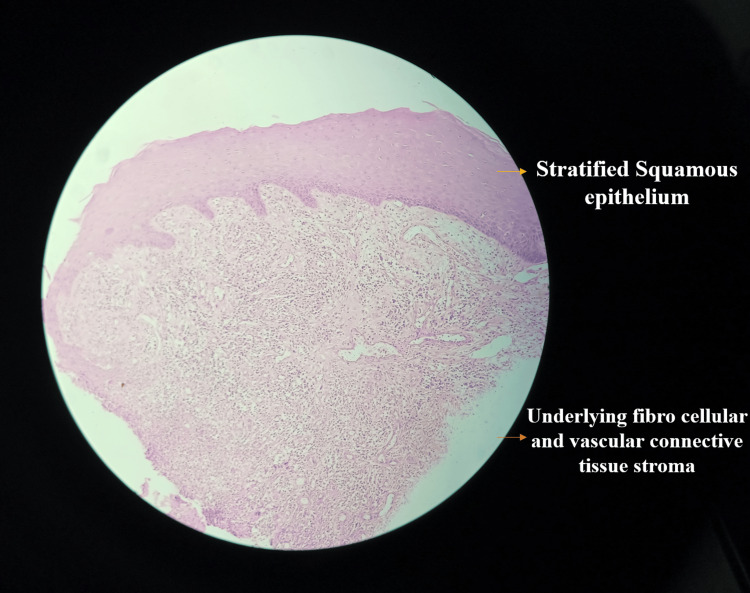
Histopathological examination under low magnification revealed epithelium and highly vascular connective tissue stroma infiltration

**Figure 9:**
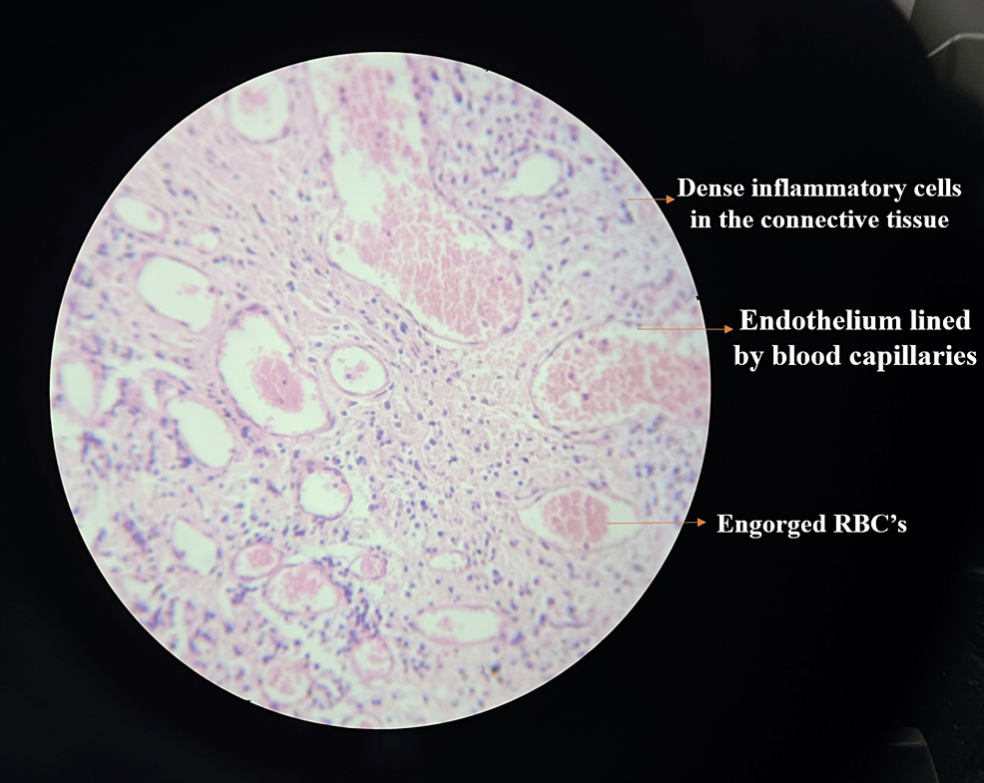
Histopathological examination under high magnification shows dense infiltration of inflammatory cells and blood capillaries engorged with RBCs

Under low magnification (4x to 10x), the H&E-stained tissue section showed overlying keratinised stratified squamous epithelium and underlying connective tissue stroma. The connective tissue stroma was highly vascular with dense inflammatory cell infiltrate, whereas, at higher magnification, the connective tissue stroma showed endothelium-lined blood capillaries engorged with RBCs. Dense inflammatory cell infiltration was seen predominantly in plasma lymphocytes, as shown in Figure 8 (low magnification) and Figure 9 (high magnification). By clinicopathologic correlation, features suggestive of PG confirmed the same.

## Discussion

The gingiva accounts for about 75% of PGs in the oral cavity. Lesions are more common on the facial aspect of the gingiva than on the lingual/palatal surface, and they can affect both sides of the gingiva, including the interdental papilla [6]. Usually, the lesion is a smooth, pedunculated, raised, sessile vascular mass with a warty, lobulated, or soft surface. It frequently develops ulcers and bleeds easily, either spontaneously or in reaction to little trauma. Lesions that have been surgically excised may return because they are not fully encapsulated, making it difficult for the surgeon to identify their boundaries and perform an adequate excision [7].

When excising a PG, extreme care should always be taken to scale the adjacent teeth and ensure that they are free of calculus since the calculus may act as an irritation that leads to the recurrence of the lesion [8]. Careful microscopic examination of the excised PG will almost invariably reveal fragments of calculus on the inner surface of the lesion adjacent to the teeth. According to histopathology, lobular aggregates of capillary-sized vessels make up lobular capillary hemangiomas, where a central feeder vessel is present in each lobule [9]. A granuloma, on the other hand, is characterised by a collection of macrophages, either surrounded by an inflammatory infiltrate or not [10]. There is no chance of developing malignant changes with these lesions. Nevertheless, they could require treatment for these reasons since they don't relieve on their own and can bleed, ulcerate, or cause unaesthetic appearances [10]. Simpler, non-invasive treatment protocols that eliminate lesions while maintaining and enhancing the mucogingival complex should also be taken into consideration [8,11].

## Conclusions

This case report demonstrates the surgical excision of a PG assisted with electrocautery, which maintained hemostasis and was discovered to be a simple, efficient, and reliable treatment approach. This painless approach by electrocautery gives patients aesthetic and functional outcomes that fulfil their demands. Surgical intervention and long-term follow-up results revealed satisfactory healing and no recurrence in the patient.
